# Magnesium and Potassium Supplementation for Systolic Blood Pressure Reduction in the General Normotensive Population: A Systematic Review and Subgroup Meta-Analysis for Optimal Dosage and Treatment Length

**DOI:** 10.3390/nu16213617

**Published:** 2024-10-24

**Authors:** Benjamin J. Behers, Brett M. Behers, Christoph A. Stephenson-Moe, Ian A. Vargas, Zhuo Meng, Anthony J. Thompson, Julian Melchor, Caroline N. Wojtas, Manuel A. Rosario, Joel F. Baker, Alexander C. Deevers, Roxann W. Mouratidis, Michael J. Sweeney

**Affiliations:** 1 Department of Clinical Sciences, Florida State University College of Medicine, Tallahassee, FL 32304, USA; cstephensonmoe@med.fsu.edu (C.A.S.-M.); ajt16b@med.fsu.edu (A.J.T.); jm15v@med.fsu.edu (J.M.); roxann.mouratidis@med.fsu.edu (R.W.M.); michael.sweeney@med.fsu.edu (M.J.S.); 2 Florida State University Internal Medicine Residency at Sarasota Memorial Hospital, Sarasota, FL 34239, USA; ian-vargas@smh.com (I.A.V.); caroline-wojtas@smh.com (C.N.W.); manuel-rosarioespinal@smh.com (M.A.R.); joel-baker@smh.com (J.F.B.); 3 Department of Clinical Research, University of South Florida College of Medicine, Tampa, FL 33602, USA; brettbehers@usf.edu; 4 Department of Statistics, Florida State University, Tallahassee, FL 32306, USA; zmeng@fsu.edu; 5 Department of Clinical Research, University of Florida, Gainesville, FL 32603, USA; a.deevers@ufl.edu

**Keywords:** magnesium, potassium, nutraceuticals, heart disease, stroke, cardiovascular disease

## Abstract

Background/Objectives: Studies have shown that consistent reductions of 2 mm Hg in systolic blood pressure (SBP) for the general normotensive population can result in significant decreases in mortality from heart disease and stroke. The purpose of this meta-analysis was to determine the optimal dose and duration of treatment for magnesium and potassium supplementation, having previously discovered that both reduce SBP by −2.79 and −2.10 mm Hg, respectively. Methods: Placebo-controlled, randomized clinical trials examining the effects of magnesium and potassium supplementation on SBP were identified. Pairwise meta-analyses with subgroups for dosage and treatment duration were run. Results: Magnesium at dosages of ≤360 mg/day and durations greater than 3 months reduced SBP by −3.03 and −4.31 mm Hg, respectively. Potassium at dosages of ≤60 mmol/day and durations greater than 1 month reduced SBP by −2.34 and −2.80 mm Hg, respectively. Conclusions: Both supplements demonstrated greater reductions in SBP for the general population at lower dosages and longer treatment durations. Future studies are needed to validate these findings and provide tailored recommendations. These studies could investigate varying dosages over long-term follow-up to provide robust data on optimal dosages and treatment durations, as our findings were limited due to reliance on previously published trials.

## 1. Introduction

Cardiovascular disease (CVD) is the leading cause of morbidity and mortality globally and has been identified as an important contributor to the cost of medical care [1]. CVD is an umbrella term that encompasses multiple disease processes, the four most important of which are coronary artery disease (CAD), cerebrovascular disease (CVD), peripheral artery disease (PAD), and aortic atherosclerosis, representing a mixture of diseases and morphological processes [2]. The vast majority of the mortalities associated with CVD are from coronary artery disease and cerebrovascular disease, causing myocardial infarction and stroke, respectively. The main pathophysiological drivers of CVD are atherosclerosis, CAD, and arterial hypertension [3]. In this article, we focus on the role that arterial hypertension plays in the development of CVD, which is via three primary mechanisms: matrix metalloproteinases, immune system activation, and oxidative stress [3].

Hypertension has been associated with remodeling of the vascular extracellular matrix (ECM) by matrix metalloproteinases (MMPs) and their tissue inhibitors (TIMPs) [3]. MMPs are a family of zinc-dependent endopeptidases involved in tissue repair, cell mobility, angiogenesis, cellular proliferation, cell migration, and apoptosis, amongst other roles [3]. Dysfunction of their activity can cause tissue destruction, fibrosis, and matrix weakening [4]. Alternatively, hypertension may lead to an imbalance between MMPs and their inhibitors (TIMPs), leading to excessive degradation of the ECM and compromise of the vascular wall [5]. In short, MMPs and TIMPs are involved in maintenance of the vascular ECM and hypertension can alter their function, leading to vascular tissue disruption.

The development of arterial hypertension has been linked to activation of cells in both the innate and adaptive immune system, as well as elevations in inflammatory markers, cytokines, and antibodies [3]. Macrophages, which are the main effector cells of the innate immune system, have been shown to play a role in arterial hypertension, with elevated levels seen in hypertensives compared to normotensives [3]. Furthermore, monocytes can be activated by elevated levels of angiotensin II (Ang II), as seen in hypertension, and cause subendothelial infiltration and increase the risk of atherosclerotic complications [6]. Elevations in pro-inflammatory cytokines, such as interleukin (IL)-6, IL-1β, IL-1α, IL-18, IL-2, IL-8, tumor necrosis factor (TNF)-α, interferon (IFN)-γ, C-reactive protein (CRP), and monocyte chemoattractant protein (MCP)-1, have been associated with arterial hypertension [7]. Furthermore, decreases in IL-10, an anti-inflammatory cytokine, have also been described [7]. In summary, arterial hypertension leads to activation of both the innate and adaptive immune systems, leading to inflammation that predisposes to the development of CVD.

The last mechanism by which arterial hypertension leads to the development of CVD is via oxidative stress causing endothelial dysfunction and resultant vascular remodeling. Oxidative stress, which results in either increased reactive oxygen species (ROS) production or decreases in antioxidant defense, works alongside inflammatory responses in arterial hypertension [8,9]. Vascular injury from ROS is mediated through promotion of vascular smooth muscle cell growth, extracellular matrix deposition, activation of matrix metalloproteinases, inflammation, endothelial dysfunction, and increased vascular tone [10]. Furthermore, the ROS-producing NADPH oxidase (NOX) enzymes have been implicated in the vascular remodeling seen during arterial hypertension [3]. In fact, overproduction of NOX1 in vascular smooth muscle is responsible for the hypertrophic and hypertensive responses to Ang II, thus participating in the development of cardiovascular pathologies [11]. All in all, arterial hypertension is associated with increased production of ROS, which directly cause endothelial damage and potentially CVD.

While uncontrolled hypertension is an obvious risk factor for heart disease and stroke, increased risks of mortality from these causes can also be seen across a range of blood pressure (BP) values. The American College of Cardiology–American Heart Association (ACC/AHA) task force and the seventh report of the Joint National Committee on Prevention, Detection, Evaluation, and Treatment of High Blood Pressure (JNC 7) classify normal blood pressure as <120/<80 mm Hg, acknowledging the risk of cardiovascular disease at higher readings [12,13]. However, studies have determined the optimal BP to be 110–115/70–75 mm Hg, with an increased risk of mortality from heart disease and stroke seen with higher readings [14,15]. Further, consistent reductions of 2 mm Hg in systolic blood pressure (SBP) for the general normotensive population can result in significant decreases in mortality from heart disease and stroke, as well as morbidity from disabling strokes [15]. This raises the question of whether similar pathophysiological mechanisms to those seen with overt arterial hypertension occur at BP above the optimal range.

Although there have been efforts to lower blood pressure amongst hypertensive patients due to the aforementioned risks, considerably less attention has been directed towards lowering blood pressure amongst normotensive patients. This was the primary motivation for our 2023 meta-analysis, which explored the potential for six nutraceuticals to lower blood pressure amongst otherwise healthy normotensive patients [16]. We found calcium and magnesium provided statistically significant reductions in both SBP and diastolic blood pressure (DBP) of 1.37/1.63 and 2.79/1.56 mm Hg, respectively [16]. Potassium and vitamin E provided only statistically significant reductions in SBP of 2.10 and 1.76 mm Hg, respectively, while vitamins C and D provided no statistically significant reductions [16]. As hypothesized, our reductions were less than those found in other meta-analyses due to our controlling for a normotensive population.

We also explored the mechanisms by which these nutrients lower BP. Vitamins C and E prevent endothelial dysfunction through their antioxidant effects and enhancement of nitric oxide pathways [16]. Vitamin D has been shown to regulate the renin–angiotensin–aldosterone system (RAAS), with lower levels leading to increased activity of this system [16]. Calcium regulates BP through the parathyroid and vitamin D systems and the RAAS, with lower levels of calcium leading to vasoconstriction, increased peripheral vascular resistance, and ultimately increased BP [16]. Magnesium can affect the release of nitric oxide, thus acting on calcium concentrations and causing alteration of smooth muscle tone and endothelial dysfunction when magnesium levels are low [16]. Potassium directly affects BP, with increased levels causing decreased reabsorption of sodium and chloride, as well as decreased RAAS activity [16].

Given the clinical significance of SBP reductions of 2 mm Hg, in addition to the global burden of CVD, we decided to further explore the potential of magnesium and potassium. The objective of this study was to investigate optimal dosage and treatment duration of magnesium and potassium supplementation for SBP reduction in the general population through subgroup meta-analyses. It was hypothesized that higher dosages over longer treatment durations would produce the greatest SBP reductions. We feel it is important to elucidate these data to provide guidance on how to take these supplements most effectively because of their potential cardiovascular health benefits, as well as the lack of attention paid to the health optimization of the normotensive population.

## 2. Methods

This study adhered to the Preferred Reporting Items for Systematic Reviews and Meta-Analyses (PRISMA) statement [17]. Our methodology was adapted from our original meta-analysis to be specific for magnesium and potassium supplementation [16].

### 2.1. Eligibility Criteria

Inclusion criteria were: (1) placebo-controlled randomized controlled trials (RCTs) of magnesium or potassium supplementation that were published in English; (2) focused on a general adult population; (3) reported SBP effects; and (4) lasted at least two weeks. A language-restrictive meta-analysis was performed due to all authors being solely English-speaking, so we lacked a way to validate accuracy if we elected to translate articles published in another language. We defined “general adult population” as consisting of participants over the age of 18 with less than half of participants having a common medical condition. For instance, if 51% of study participants had hyperlipidemia or CAD, then that study was excluded. We also excluded studies that had participants whose mean BP was in the hypertensive range at screening, even if they had not been formally diagnosed with hypertension.

Exclusion criteria were: (1) other study types aside from RCTs; (2) were not published in English; (3) lack of supplement dosing; (4) did not focus on a general adult population; (5) had pediatric or pregnant participants; (6) provided insufficient data on SBP changes; and/or (7) lasted less than two weeks.

### 2.2. Information Sources and Search Strategy

Cochrane, Embase, Medline (PubMed), and Web of Science were systematically searched for placebo-controlled RCTs examining the effects of magnesium and potassium supplementation on BP. However, as mentioned, this search was part of a broader meta-analysis and thus includes additional supplements. The literature search strings can be seen in Table 1.

### 2.3. Selection Process

Studies identified through the systematic search were imported into Covidence, systematic review management software [18]. Two authors independently screened all studies by their title and abstract. After excluding irrelevant studies, full-text articles were evaluated for their adherence to the eligibility criteria by the same two authors. A third author was available to solve disagreements during the screening process that could not be solved through discussion.

### 2.4. Data Collection Process and Data Items

Two authors independently extracted the basic characteristics of included studies, with subsequent review by the first author. This included the last name of the first author, year of publication, country where the trial was performed, whether it was a parallel or cross-over trial, population included in the trial, age of participants, magnesium and potassium dosing, and trial duration. The first author extracted SBP data for all arms of the trial, with verification for accuracy by two other authors.

### 2.5. Study Risk-of-Bias Assessment

Quality assessment was performed using the Risk of Bias 2 tool from the *Cochrane Handbook for Systematic Reviews of Interventions* (Cochrane Handbook) [19]. Risk of bias was examined based on five domains: (1) the randomization process; (2) deviations from planned interventions; (3) missing outcome data; (4) the method for measuring the outcome; and (5) selection of the reported outcome [19]. Two authors independently performed this assessment for each included study, with a third author solving any disagreements. Studies were deemed to be at overall high risk of bias if they had any domains found to be at high risk or three domains having some concerns. When studies had two domains with some concerns, they had some concerns for bias overall.

### 2.6. Effect Measures and Synthesis Methods

Change in SBP between the supplement (magnesium or potassium) and placebo groups was the primary endpoint. The standard error (SE) of this change was also extracted. When not reported, SE was calculated using other data provided, such as 95% confidence intervals (CIs). Missing standard deviations were imputed using a correlation coefficient of 0.7. Subgroup analyses were performed based on dosage and treatment duration. The cutoffs for these subgroups were arbitrarily determined by identifying a dosage or duration that would produce a roughly equal number of studies in each subgroup.

Overall effect sizes and their 95% CIs were reported for each subgroup. Both common- and random-effect models were run, with the level of heterogeneity using the Q-statistic at the 0.1 significance level determining which value to report [20]. Percentage of variability accounted for by between-study variation was determined by the *I*^2^ statistic, and this variation was estimated using the restricted maximum likelihood (REML) method [21,22]. Sensitivity analyses were performed for parallel and cross-over studies according to Chapters 6 and 23 of the Cochrane Handbook [23,24]. R 4.3.0 and the R package “meta” were used to conduct these analyses [25].

### 2.7. Reporting Bias Assessment

Egger’s regression was used to assess publication bias using a significance level of 0.1 [26]. Visualization of potential publication bias was achieved using contour-enhanced funnel plots [27,28].

## 3. Results

### 3.1. Study Selection

**Figure 1:**
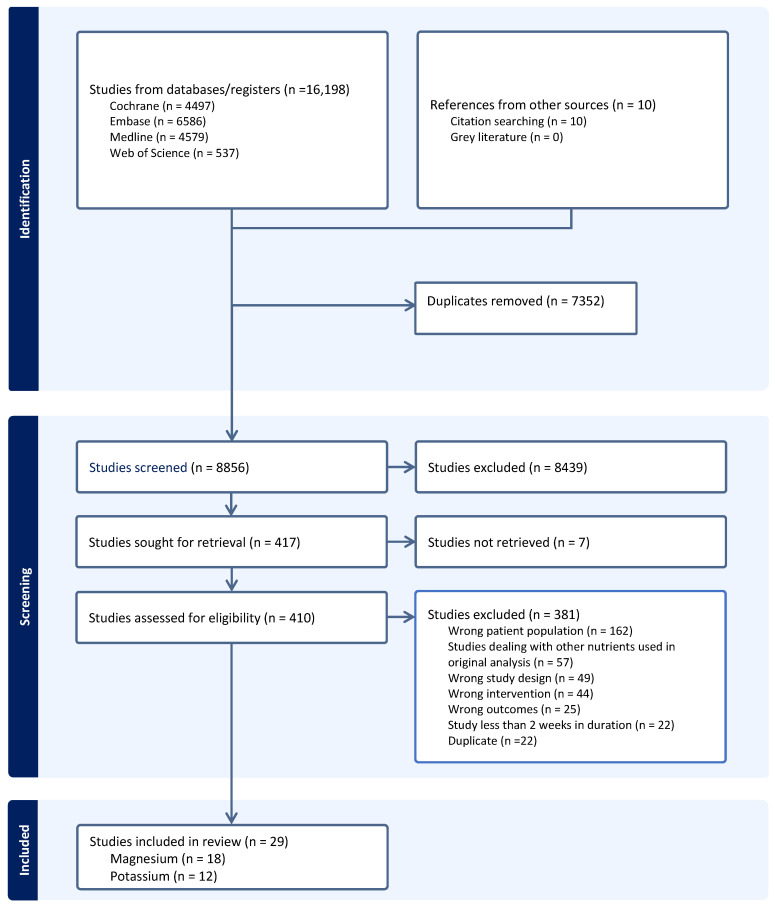
The PRISMA flowchart detailing literature search and selection.

As mentioned, the literature search was performed for a previous study that investigated six nutraceuticals, including magnesium and potassium. This search yielded 16,198 total articles across the four databases. In sum, 10 additional studies were identified by hand-searching the reference lists of included studies, for a total of 16,208 articles identified. After 7352 duplicates were subsequently excluded by Covidence, the remaining 8856 articles were screened using their title and abstracts. This resulted in the exclusion of 8439 studies, with the full texts being sought for the remaining 417 studies. Seven of these studies were unable to be retrieved due to them only being an abstract (*n* = 3), not obtainable in English (*n* = 2), or the full text being otherwise unobtainable (*n* = 2). Of the 410 full texts that were assessed for eligibility, 29 studies were ultimately included in this analysis. There were 18 eligible studies with magnesium supplementation and 12 with potassium supplementation, as one study had trial arms for each [29]. The study selection process is summarized by a PRISMA flowchart in Figure 1.

### 3.2. Study Characteristics

A total of 29 studies were included in this analysis, with 18 on magnesium supplementation and 12 on potassium supplementation [29,30,31,32,33,34,35,36,37,38,39,40,41,42,43,44,45,46,47,48,49,50,51,52,53,54,55,56,57].

The 18 studies on magnesium supplementation included 1529 participants [29,30,31,32,33,34,35,36,37,38,39,40,41,42,43,44,45,46]. They were performed in 11 different countries, with Mexico (n = 5) and the United States of America (n = 3) being the most common. Most of the studies were parallel trials (n = 14), while the remaining were cross-over trials (n = 4). The breakdown of patient populations was as follows: healthy (n = 8), general (n = 6), and overweight (n = 4). The mean age of participants ranged from 21.2 to 64.6 years. Blood pressure was measured using resting office readings in all but one trial, with it using 24 h ambulatory readings [29]. Magnesium dosage ranged from 212 to 497.5 milligrams per day (mg/day), while treatment duration ranged from 4 weeks to 6 months. The basic characteristics of included magnesium studies can be found in Table 2.

The 12 studies on potassium supplementation consisted of 1065 participants [29,47,48,49,50,51,52,53,54,55,56,57]. They were performed in four countries, with the United States of America (n = 5) and England (n = 4) being the most common. Trial types were almost evenly split between parallel (n = 7) and cross-over (n = 5) designs. The patient populations of these trials were also almost evenly split between healthy (n = 7) and general (n = 5). The mean age of participants ranged from 23.7 to 56 years. Blood pressure was measured using 24 h ambulatory readings in three trials [29,48,51]. Potassium dosage ranged from 24 to 100 millimoles per day (mmol/day), while trial duration ranged from 3 weeks to 6 months. The basic characteristics of included potassium studies can be found in Table 3.

### 3.3. Risk of Bias in Studies

**Figure 2:**
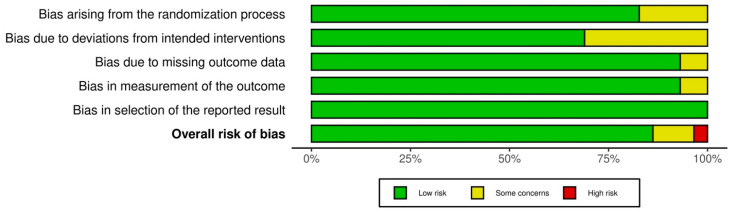
Graphical representation of the risk of bias across all five domains, as well as the overall judgment, for all included studies.

Domain 2 (bias arising from deviations from the intended interventions) had the most studies, with “Some concerns” about risk of bias in nine [37,40,41,42,44,47,52,54,55]. Domain 1 (bias arising from the randomization process) was the next most implicated, with five [29,44,45,53,55]. Domains 3 (bias arising from missing outcome data) and 4 (bias arising from the method of measuring the outcome) each had two studies with “Some concerns” [32,47,54,55], while domain 5 (bias arising from selection of the reported result) had none. Across the 29 studies, the overall risk of bias was determined to be low in 25 of them, some concerns in 3, and high in 1. A study on magnesium supplementation accounted for one of the studies with some concerns [44]. Studies on potassium supplementation accounted for the other two studies with some concerns [47,54], as well as the only study with high risk of bias [55]. A graphical representation of the risk of bias across all five domains, as well as the overall judgment, can be found in Figure 2.

### 3.4. Results of Syntheses/Statistical Analysis

#### 3.4.1. Magnesium

**Figure 3:**
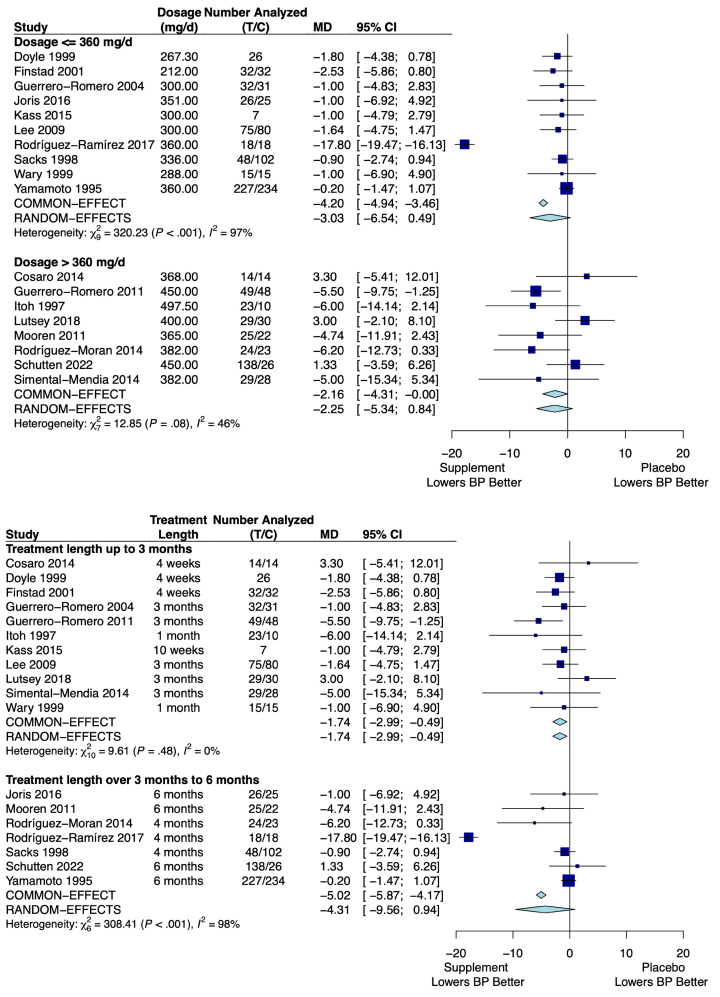
Forest plots showing the mean difference of change in systolic blood pressure of magnesium supplementation versus placebo by subgroup for dosage and treatment length. T represents the sample size of the magnesium group and C represents the sample size of the control (placebo) group. References: [29,30,31,32,33,34,35,36,37,38,39,40,41,42,43,44,45,46].

Eighteen trials of magnesium supplementation met the inclusion criteria, consisting of 1529 participants. The pooled results for the difference in the change in SBP for the magnesium group versus placebo for each subgroup are shown in Figure 3.

Ten trials with 1011 participants dealt with magnesium supplementation of ≤360 mg/day, yielding a mean difference (MD) in SBP of −3.03 mm Hg (95% CI: −6.54, 0.49) using the random-effect model due to high heterogeneity (*I*^2^ = 97%; *p*-value for the *Q*-statistic: <0.001). The other eight trials, consisting of 518 participants, investigated dosages above 360 mg/day and had an MD in SBP of −2.25 mm Hg (95% CI: −5.34, 0.84) using the random-effect model due to high heterogeneity (*I*^2^ = 46%; *p*-value for the *Q*-statistic: 0.08).

Eleven trials with 573 participants dealt with magnesium supplementation for a duration of up to three months, yielding an MD in SBP of −1.74 mm Hg (95% CI: −2.99, −0.49) using the common-effect model due to low heterogeneity (*I*^2^ = 0%; *p*-value for the *Q*-statistic: 0.48). The other seven trials, consisting of 956 participants, investigated magnesium supplementation for 3 to 6 months and had an MD in SBP of −4.31 mm Hg (95% CI: −9.56, 0.94) using the random-effect model due to high heterogeneity (*I*^2^ = 98%; *p*-value for the *Q*-statistic: <0.001).

These results are summarized in Table 4.

#### 3.4.2. Potassium

**Figure 4:**
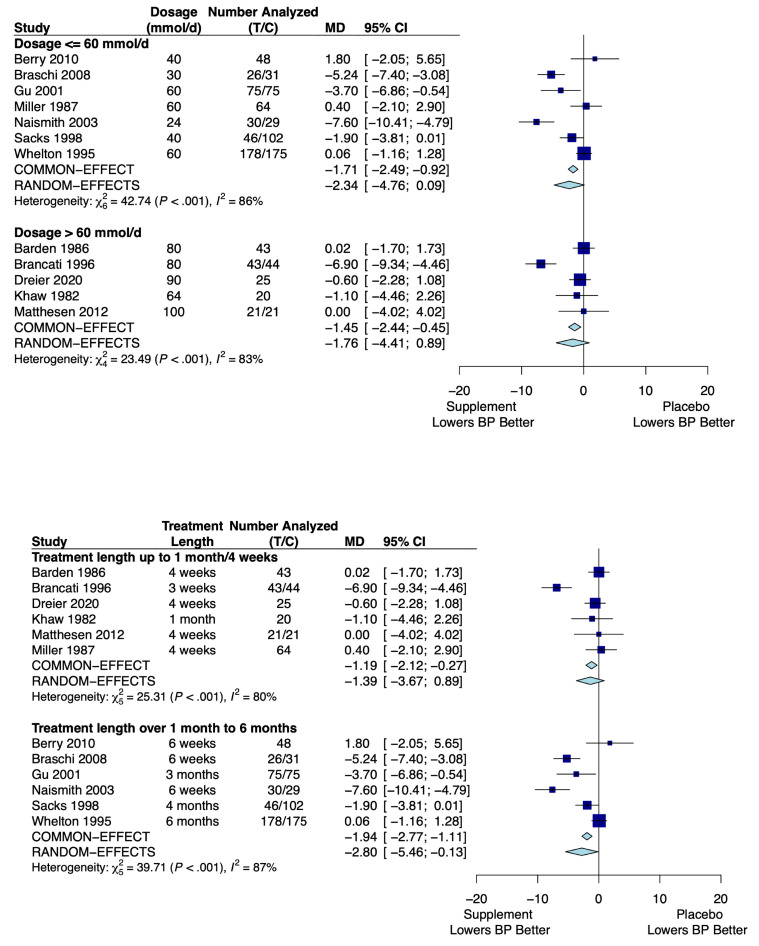
Forest plots showing the mean difference of change in systolic blood pressure of potassium supplementation versus placebo by subgroup for dosage and treatment length. T represents the sample size of the potassium group and C represents the sample size of the control (placebo) group. References: [29,47,48,49,50,51,52,53,54,55,56,57].

Twelve trials of potassium supplementation met the inclusion criteria, consisting of 1065 participants. The pooled results for the difference in the change in SBP for the potassium group versus placebo for each subgroup are shown in Figure 4.

Seven trials with 879 participants dealt with potassium dosages of ≤60 mmol/day, yielding an MD in SBP of −2.34 mm Hg (95% CI: −4.76, 0.09) using the random-effect model due to high heterogeneity (*I*^2^ = 86%; *p*-value for the *Q*-statistic: <0.001). The other five trials, consisting of 186 participants, investigated dosages over 60 mmol/day and had an MD in SBP of −1.76 mm Hg (95% CI: −4.41, 0.89) using the random-effect model due to high heterogeneity (*I*^2^ = 83%; *p*-value for the *Q*-statistic: <0.001).

Six trials with 250 participants dealt with potassium supplementation up to 1 month, yielding a MD in SBP of −1.39 mm Hg (95% CI: −3.67, 0.89) using the random-effects model due to high heterogeneity (*I*^2^ = 80%; *p*-value for the *Q*-statistic: <0.001). The other six trials, consisting of 815 participants, investigated potassium supplementation for 1 to 6 months and had a MD in SBP of −2.80 mm Hg (95% CI: −5.46, −0.13) using the random-effects model due to high heterogeneity (*I*^2^ = 87%; *p*-value for the *Q*-statistic: <0.001).

These results are summarized in Table 5.

### 3.5. Sensitivity Analysis

The results of the sensitivity analysis are summarized in Table 6. Overall, no analyses became nonsignificant when a smaller correlation coefficient was used to impute standard deviations of change-from-baseline scores. This was evidenced by none of the CIs including zero. However, the use of 0.5 and 0.7 for potassium supplementation resulted in the presence of publication bias.

### 3.6. Publication Bias

**Figure 5:**
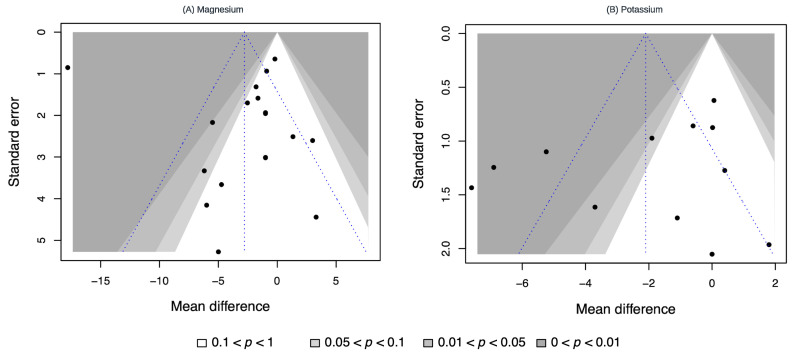
Contour-enhanced funnel plots of the meta-analyses investigating magnesium and potassium supplementation for systolic blood pressure reduction.

Contour-enhanced funnel plots for the effects of magnesium and potassium on SBP with their effect sizes on the horizontal axis and SEs on the vertical axis can be seen in Figure 5. The lack of asymmetry in these plots indicates no potential publication bias associated with these analyses. Further, Egger’s regression did not yield any significant results to indicate publication bias.

## 4. Discussion

Our results indicate that greater reductions in SBP were seen at lower dosages and longer treatment durations for both magnesium and potassium supplementation. Magnesium at dosages of ≤360 mg/day and durations greater than 3 months reduced SBP by 3.03 and 4.31 mm Hg, respectively. Potassium at dosages of ≤60 mmol/day and durations greater than 1 month reduced SBP by 2.34 and 2.80 mm Hg, respectively. Our findings partially supported our hypothesis, as longer treatment durations yielded greater reductions in SBP. However, lower dosages were also associated with greater reductions in SBP, which we did not predict. These results demonstrate how the general population can use these supplements most effectively by guiding optimal dosage and treatment duration. Further, the greater reductions in SBP with longer treatment durations hold promise that these supplements can achieve consistent reductions of 2 mm Hg in SBP and lead to reduction in the risk of mortality from heart disease and stroke for the general population. Our study is also important because it focuses on the normotensive population, which can still be at risk of suffering from cardiovascular complications, but are studied far less than their hypertensive counterparts.

Interestingly, lower dosages of both supplements appear to be more effective in reducing SBP than higher ones. To further understand the discrepancy between dosing and BP reduction, we compared our findings with the most recently published meta-analyses investigating the effect of magnesium and potassium supplementation on blood pressure. A meta-analysis on magnesium supplementation divided these trials into three subgroups for both dosage and treatment length [58]. They found the greatest reduction in SBP were 5.33 mm Hg and 2.82 mm Hg at dosages <300 mg/day and treatment lengths of 30–89 days, respectively [58]. However, an older meta-analysis noted a dose-dependent relationship between magnesium supplementation and SBP reduction, with reductions of 4.3 mm Hg in SBP for each 10 mmol/day increase in magnesium dose [59]. Therefore, no consensus was obtained on either dosage or treatment length between our findings and these prior studies. It should be noted that both prior studies included hypertensive subjects and likely obtained greater reductions as a result, as opposed to controlling for a general normotensive population as we did.

The most recent meta-analysis investigating the effect of potassium supplementation on blood pressure was also a dose–response meta-analysis, which allowed for better insight into optimal dosage [60]. Their findings suggest a U-shaped response between potassium dosage and SBP reduction, with lower dosages resulting in a greater reduction and higher dosages producing a lesser effect, even increasing SBP after a dosage of 80 mmol/day [60]. Furthermore, they ran a subgroup analysis by hypertension status and found that normotensive participants experienced a lesser reduction in SBP and were more sensitive to higher dosages of potassium compared to the hypertensive group [60]. In fact, the greatest reductions in SBP. around 2 mm Hg. amongst normotensives were seen with potassium dosages of 20–30 mmol/days, while the hypertensive effect of potassium started at 60 mmol/day [60]. However, another meta-analysis reported contradicting results with higher dosages of potassium, specifically ≥100 mmol/day, yielding the greatest reduction in SBP of 4.9 mm Hg [61]. Given that this study did not control for hypertension status, we believe that lower dosages of potassium supplementation may be both safer and more effective for the general normotensive population. With that in mind, future studies should explore this further, potentially through a dose–response analysis with a variety of lower dosages with large samples.

It is important to explore the mechanisms by which magnesium and potassium are thought to lower BP. Magnesium is a predominantly intracellular cation that is involved in a wide range of cellular processes and metabolic reactions. There are several hypothesized mechanisms for how magnesium lowers blood pressure, ultimately leading to either reduced total peripheral resistance or decreased cardiac output [62]. Reduced total peripheral resistance occurs from either decreased vascular stiffness or through vasodilation of the blood vessels [62]. Decreased vascular stiffness is achieved via increased magnesium transporter transient receptor potential melastatin 7 (TRPM7) activity, resulting in reduced vascular calcification [63]. On the other hand, vasodilation of the blood vessels occurs through either direct inhibition of calcium channels or via production of nitric oxide and prostacyclin by the endothelium [64,65,66]. Alternatively, the decreased cardiac output is mediated by decreased reabsorption of sodium [62]. This occurs through either increased atrial natriuretic peptide (ANP) levels or reduced intracellular calcium that causes a resultant reduction in Ang II and thus aldosterone [67,68].

Similarly, potassium is another primarily intracellular cation that plays a fundamental role in regulating ionic and osmotic gradients across cell membranes. There are numerous ways in which potassium exerts its effect on blood pressure, but the predominant mechanism is thought to be via sodium–potassium ATPase-mediated vascular smooth muscle hyperpolarization [69]. This role of potassium in maintaining electrical potential is thought not only to exhibit vasodilatory properties, including in the coronary circulation, but also to modulate salt sensitivity through its effect on the sodium chloride co-transporter (NCC) in the distal convoluted tubule (DCT) [70]. Increases in dietary potassium intake have been shown to suppress this activity, leading to reduced salt reabsorption and thus lower blood pressure. Other mechanisms include increased natriuresis, modulation of baroreceptor sensitivity, reduced vasoconstrictive sensitivity to norepinephrine and Ang II, and decreases in NADPH oxidase, oxidative stress, and inflammation [71]. As mentioned, NADPH oxidase and oxidative stress are pathophysiological mechanisms of hypertension linked to the development of cardiovascular disease.

Our study is not without limitations, the greatest of which is the high heterogeneity observed across most analyses, a common limitation amongst meta-analyses. We hope that our use of the random-effect model to convey these results helps to mitigate this; however, use of this model can result in wider confidence intervals with the potential of incorrectly deeming results insignificant [72]. Our results must thus be interpreted with caution. The arbitrary method we used to develop our subgroup cutoffs poses another limitation. As mentioned, we chose these values by seeking cutoffs that would roughly halve the number of studies, with the hopes of yielding large enough samples for analysis. Furthermore, the longest trials that met the criteria for our study were only 6 months in duration, and inclusion into the highest subgroup for treatment duration started at 3 months and 1 month for magnesium and potassium, respectively. These relatively short trials make commenting on long-term efficacy challenging. This reliance on previously published trials for our data did not allow us to adequately assess specific dosages over specific treatment durations either. Future studies could pursue a dose–response analysis to better assess optimal dosage over longer trials, given that greater reductions were seen with longer treatment durations for both supplements. Future studies could also investigate whether there are any additive or synergistic effects with magnesium and potassium co-supplementation. These studies would be better equipped to investigate and determine long-term efficacy and safety. Our study did not control for the baseline characteristics of included participants for each trial either. For instance, while we labeled populations as “healthy” or “general,” we did not account for possible variations between these populations that could affect their response to supplementation. Variations include baseline levels of these nutrients, dietary intake, and even comorbidities, despite our strict inclusion criteria. A meta-regression accounting for these differences, including baseline BP variations, would make this analysis stronger. Ultimately, despite not being able to report the exact ideal dosage or treatment duration, our study provides valuable insight into where patients or future researchers should start.

## 5. Conclusions

Ultimately, these supplements seem promising to achieve optimal BP control in the general normotensive population and reduce their risk of mortality from myocardial infarction or stroke. While both magnesium and potassium supplementation produced greater SBP reductions over longer treatment durations, they also achieved greater reductions at lower dosages. Future studies are necessary to determine exact dosage and treatment length, as well as long-term efficacy and safety.

## Figures and Tables

**Table 1:** Literature search strings and results across the four databases. Boolean operators and keyword expansions were specifically tailored to each database.

Database	Search String
Cochrane Date run: 22 July 2022 Number of results: 4497	([mh “ascorbic acid”] OR [mh “vitamin D”] OR [mh “vitamin E”] OR [mh “Vitamin B Complex”] OR “vitamin C”:ti,ab OR “ascorbic acid”:ti,ab OR “vitamin D”:ti,ab OR “vitamin E”:ti,ab OR “vitamin B”:ti,ab OR (“B” NEXT vitamin*):ti,ab OR [mh calcium] OR [mh magnesium] OR [mh potassium] OR calcium:ti,ab OR magnesium:ti,ab OR potassium:ti,ab) AND ([mh “blood pressure”] OR “blood pressure”:ti,ab) “Randomized controlled trial”:pt OR “controlled clinical trial”:pt
Embase Date run: 22 July 2022 Number of results: 6585	(“ascorbic acid”/exp OR “vitamin D”/exp OR “vitamin E”/exp OR “Vitamin B Complex”/exp OR “vitamin C”:ti,ab OR “ascorbic acid”:ti,ab OR “vitamin D”:ti,ab OR “vitamin E”:ti,ab OR “vitamin B”:ti,ab OR “B vitamin*”:ti,ab OR calcium/exp OR magnesium/exp OR potassium/exp OR calcium:ti,ab OR magnesium:ti,ab OR potassium:ti,ab) AND (“blood pressure”/exp OR “blood pressure”:ti,ab) “randomized controlled trial” OR “controlled clinical trial”
Medline (PubMed) Date run: 22 July 2022 Number of results: 4579	(ascorbic acid[MeSH] OR vitamin D[MeSH] OR vitamin E[MeSH] OR Vitamin B Complex[MeSH] OR “vitamin C”[tiab] OR “ascorbic acid”[tiab] OR “vitamin D”[tiab] OR “vitamin E”[tiab] OR “vitamin B”[tiab] OR B vitamin*[tiab] OR calcium[MeSH] OR magnesium[MeSH] OR potassium[MeSH] OR calcium[tiab] OR magnesium[tiab] OR potassium[tiab]) AND (blood pressure[MeSH] OR “blood pressure”[tiab]) Randomized controlled trial[pt] OR controlled clinical trial[pt]
Web of Science (Core Collection) Date run: 22 July 2022 Number of results: 537	(TI = “vitamin C” OR AB = “vitamin C”) OR (TI = “ascorbic acid” OR AB = “ascorbic acid”) OR (TI = “vitamin D” OR AB = “vitamin D”) OR (TI = “vitamin E” OR AB = “vitamin E”) OR (TI = “vitamin B” OR AB = “vitamin B”) OR (TI = “B vitamin*” OR AB = “B vitamin*”) OR (TI = calcium OR AB = calcium) OR (TI = magnesium OR AB = magnesium) OR (TI = potassium OR AB = potassium) AND (TI = “blood pressure” OR AB = “blood pressure”) ALL = “Randomized controlled trial” OR ALL = “controlled clinical trial”

**Table 2:** Basic characteristics of the 18 trials dealing with magnesium supplementation. Percentage of patients with baseline cardiovascular disease is included in the population column. BP was measured by resting office readings, unless denoted otherwise, with an asterisk (*) representing 24 h ambulatory readings.

Study	Country	Type of Trial	Population (% CVD)	Mean Age (Years)	Baseline BP (mm Hg)	Dosage (mg/Day)	Trial Duration
Cosaro 2014 [30]	Italy	Cross-over	Healthy (0%)	26.3	123.7/71.4	368	4 weeks
Doyle 1999 [31]	Ireland	Cross-over	Healthy (0%)	23	112.1/75.9	267.3	4 weeks
Finstad 2001 [32]	Canada	Cross-over	General (0%)	21.2	114.3/69.4	212	4 weeks
Guerrero-Romero 2004 [33]	Mexico	Parallel	Healthy (0%)	42.6	110.5/73	300	3 months
Guerrero-Romero 2011 [34]	Mexico	Parallel	General (0%)	40.6	116.6/73.8	450	3 months
Itoh 1997 [35]	Japan	Parallel	Healthy (0%)	64.6	127.3/76.1	497.5	1 month
Joris 2016 [36]	Netherlands	Parallel	Overweight (0%)	62	128/81.5	351	6 months
Kass 2015 [37]	England	Cross-over	General (0%)	40.8	118.4/81.6	300	10 weeks
Lee 2009 [38]	South Korea	Parallel	Overweight (0%)	40.1	125.7/83.4	300	3 months
Lutsey 2018 [39]	United States of America	Parallel	General (24%)	61.5	119/71	400	3 months
Mooren 2011 [40]	Germany	Parallel	Overweight (0%)	N/R	136.3/84	365	6 months
Rodriguez-Moran 2014 [41]	Mexico	Parallel	Healthy (0%)	35.6	111.8/71.5	382	4 months
Rodriguez-Ramirez 2017 [42]	Mexico	Parallel	General (0%)	51.8	127.6/77.3	360	4 months
Sacks 1998 [29]	United States of America	Parallel	Healthy (0%)	38.3	115.3/73 *	336	4 months
Schutten 2022 [43]	Netherlands	Parallel	Overweight (37.2%)	63.2	130/79	450	6 months
Simental-Mendia 2014 [44]	Mexico	Parallel	General (0%)	40.4	115.2/74.6	382	3 months
Wary 1999 [45]	France	Parallel	Healthy (0%)	23.7	126.5/76.5	288	1 month
Yamamoto 1995 [46]	United States of America	Parallel	Healthy (0%)	42.5	125/84	360	6 months

**Table 3:** Basic characteristics of the 12 trials dealing with potassium supplementation. Percentage of patients with baseline cardiovascular disease is included in the population column. BP was measured by resting office readings, unless denoted otherwise with an asterisk (*), representing 24 h ambulatory readings.

Study	Country	Type of Trial	Population (% CVD)	Mean Age (Years)	Baseline BP (mm Hg)	Dosage (mmol/Day)	Duration
Barden 1986 [47]	Australia	Cross-over	Healthy (0%)	31.5	117.5/71.4	80	4 weeks
Berry 2010 [48]	England	Cross-over	General (0%)	45.1	137/89 *	40	6 weeks
Brancati 1996 [49]	United States of America	Parallel	Healthy (0%)	48.0	126.2/77.6	80	3 weeks
Braschi 2008 [50]	England	Parallel	General (0%)	35.5	111.3/68.2	30	6 weeks
Dreier 2020 [51]	Denmark	Cross-over	Healthy (0%)	26.3	119.7/72.6 *	90	4 weeks
Gu 2001 [52]	United States of America	Parallel	General (0%)	56.0	135.5/82.3	60	3 months
Khaw 1982 [53]	England	Cross-over	Healthy (0%)	N/R	118/73.5	64	1 month
Matthesen 2012 [54]	Denmark	Cross-over	Healthy (0%)	26	116/71	100	4 weeks
Miller 1987 [55]	United States of America	Parallel	General (0%)	42	113.2/73.1	60	4 weeks
Naismith 2003 [56]	England	Parallel	General (0%)	43.1	117/73	24	6 weeks
Sacks 1998 [29]	United States of America	Parallel	Healthy (0%)	38.3	116/73 *	40	4 months
Whelton 1995 [57]	United States of America	Parallel	Healthy (0%)	23.7	121.6/80.9	60	6 months

**Table 4:** A summary of the SBP effect for each of the magnesium subgroups. SBP effect is given in MD with its 95% CI, and the larger reductions are bolded.

Subgroup	SBP Effect
**Dose ≤ 360 mg/day**	**−3.03 mm Hg (−6.54, 0.49)**
Dose > 360 mg/day	−2.25 mm Hg (−5.34, 0.84)
Treatment < 3 months	−1.74 mm Hg (−2.99, −0.49)
**Treatment > 3 months**	**−4.31 mm Hg (−9.56, 0.94)**

**Table 5:** A summary of the SBP effect for each of the potassium subgroups. SBP effect is given in MD with its 95% CI, and the larger reductions are bolded.

Subgroup	SBP Effect
**Dose ≤ 60 mmol/day**	**−2.34 mm Hg (−4.76, 0.09)**
Dose > 60 mmol/day	−1.76 mm Hg (−4.41, 0.89)
Treatment ≤ 1 month	−1.39 mm Hg (−3.67, 0.89)
**Treatment > 1 month**	**−2.80 mm Hg (−5.46, −0.13)**

**Table 6:** Results of sensitivity analyses for each supplement. Baseline-End Corr represents the correlation coefficient used for imputing missing standard deviations of change-from-baseline scores. Cross-over Corr represents the correlation coefficient used for calculating standard errors of mean differences. CE and RE represent common- and random-effect models. PB represents publication bias, where N is for no/none and Y is for yes/present.

Supplement	Baseline-End Corr	Cross-over Corr	*I*^2^ (%)	CE (95% CI)	RE (95% CI)	PB
Magnesium (18 studies)	0.7	0.9	95	−3.57 (−4.21, −2.93)	−2.78 (−5.22, −0.34)	N
		0.7	95	−3.99 (−4.69, −3.29)	−2.79 (−5.25, −0.34)	N
		0.5	95	−4.10 (−4.81, −3.38)	−2.81 (−5.28, −0.33)	N
	0.5	0.9	95	−3.64 (−4.29, −2.98)	−2.76 (−5.31, −0.21)	N
		0.7	95	−4.10 (−4.83, −3.38)	−2.77 (−5.34, −0.20)	N
		0.5	95	−4.23 (−4.97, −3.48)	−2.78 (−5.37, −0.19)	N
Potassium (12 studies)	0.7	0.9	85	−1.05 (−1.52, −0.57)	−2.03 (−3.71, −0.36)	Y
		0.7	83	−1.61 (−2.22, −0.99)	−2.10 (3.81, −0.38)	N
		0.5	83	−1.83 (−2.50, −1.16)	−2.15 (−3.90, −0.40)	N
	0.5	0.9	85	−1.05 (−1.53, −0.58)	−2.06 (−3.74, −0.37)	Y
		0.7	83	−1.62 (−2.24, −1.00)	−2.12 (−3.85, −0.39)	N
		0.5	82	−1.85 (−2.52, −1.18)	−2.18 (−3.94, −0.42)	N

## Data Availability

The original contributions presented in the study are included in the article, further inquiries can be directed to the corresponding author.
